# Nonsyndromic Generalized Radiculomegaly of Permanent Dentition: A Rare Case Report

**DOI:** 10.1155/2022/3548370

**Published:** 2022-03-29

**Authors:** Mohammed Alhussain, Naif Almosa, Hourya Alnofaie

**Affiliations:** ^1^Ministry of Health, Riyadh, Saudi Arabia; ^2^Department of Pediatric Dentistry and Orthodontics, College of Dentistry, King Saud University, Riyadh, Saudi Arabia; ^3^Division of Oral and Maxillofacial Surgery, Basic Dental Sciences Department, College of Dentistry, Princess Nourah Bint Abdulrahman University, Riyadh, Saudi Arabia

## Abstract

**Introduction:**

Radiculomegaly (marked elongation of dental roots) is a distinct dental abnormality with a major clinical significance that is closely related to oculofaciocardiodental syndrome (OFCD). Since OFCD syndrome was first identified in 1996, only a few cases of nonsyndromic/nonfamilial radiculomegaly have been reported. We report a new nonsyndromic/nonfamilial radiculomegaly case and the association of OFCD syndrome with the dental challenges. *Case Presentation*. 18-year-old medically free male presented to the screening dental clinics complaining of teeth malposition. Panoramic radiograph incidentally revealed extremely long and wide roots of almost all dentition. Apical radiographs and cone-beam computed tomography (CBCT) were taken to confirm this anomaly and to rule out any artifact. The images confirmed the excessive length of the roots. A cephalometric X-ray was performed on the patient to rule out any dentofacial deformity. History and physical examinations were negative for ocular or cardiac anomalies. Due to lack of evidence of physical signs and symptoms consistent with OFCD syndrome, genetic testing was not performed.

**Conclusion:**

Dentists need to be informed of the clinical and radiographic features of different dental anomalies, especially radiculomegaly, as it is considered one of the significant features of OFCD syndrome. Challenges related to radiculomegaly face dentists while the current literature has not yet provided a solid baseline for managing such patients. The challenges include repairing the root canals, extracting and/or moving the teeth orthodontically. Early diagnosis of the syndrome is crucial to prevent dental challenges and provide the best dental care services.

## 1. Introduction

The normal mechanism of crown and root development is a complex process, where three stages are included in the process: bud, cap, and bell stages. Hertwig's epithelial root sheath, a double layer of the epithelial sheath, provides guidance for root development when the crown is about to be completed [1]. The most common root malformations in humans arise from either developmental disorders of the root alone or disorders of radicular development as part of general tooth dysplasia [2]. Despite that, little is known about the mechanisms of root morphogenesis and elongation [3]. Hyperdontia, hypodontia, oligodontia, multiple roots, and taurodontia are all examples of abnormal tooth morphology and number [4].

Radiculomegaly, dental root gigantism, or extremely long dental roots is a rare dental anomaly affecting the roots of teeth, and canines are mostly affected. This condition has rarely been reported in the literature. The first reports of radiculomegaly [5–7] related it as a characteristic feature of the X-linked rare congenital syndrome, oculofaciocardiodental syndrome (OFCD). OFCD syndrome is an X-linked dominant trait with potential male lethality [4, 8, 9], which affects the ocular, facial, cardiac, and dental aspects, with an incidence of less than 1 in 1 million people [4, 8–11]. Among different dental anomalies that can be found with OFCD syndrome, radiculomegaly of the canines is highly related to this syndrome [4, 8, 12–14]. OFCD syndrome is characterized by heterogeneous clinical features such as dental radiculomegaly, congenital cataracts, facial dysmorphism, and congenital heart disease, which only affects females [10, 11, 15]. On the other hand, it was later identified as a defining feature of males with Klinefelter syndrome and Cockayne syndrome [16, 17].

To date, only a few nonsyndromic/nonfamilial radiculomegaly cases have been reported in the literature [8, 12, 18]. This case report aims to report a rare case of a nonsyndromic/nonfamilial generalized radiculomegaly case of an 18-year-old Saudi male patient and to investigate the association of OFCD syndrome with dental challenges.

## 2. Case Presentation

### 2.1. Patient History

An 18-year-old Saudi male patient with no relevant medical history is presented to the screening clinic at the College of Dentistry, King Saud University, Saudi Arabia. The chief complaint of the patient that he has “crooked teeth,” with an unremarkable family history of any disease.

### 2.2. Extraoral Examination

**Figure 1:**
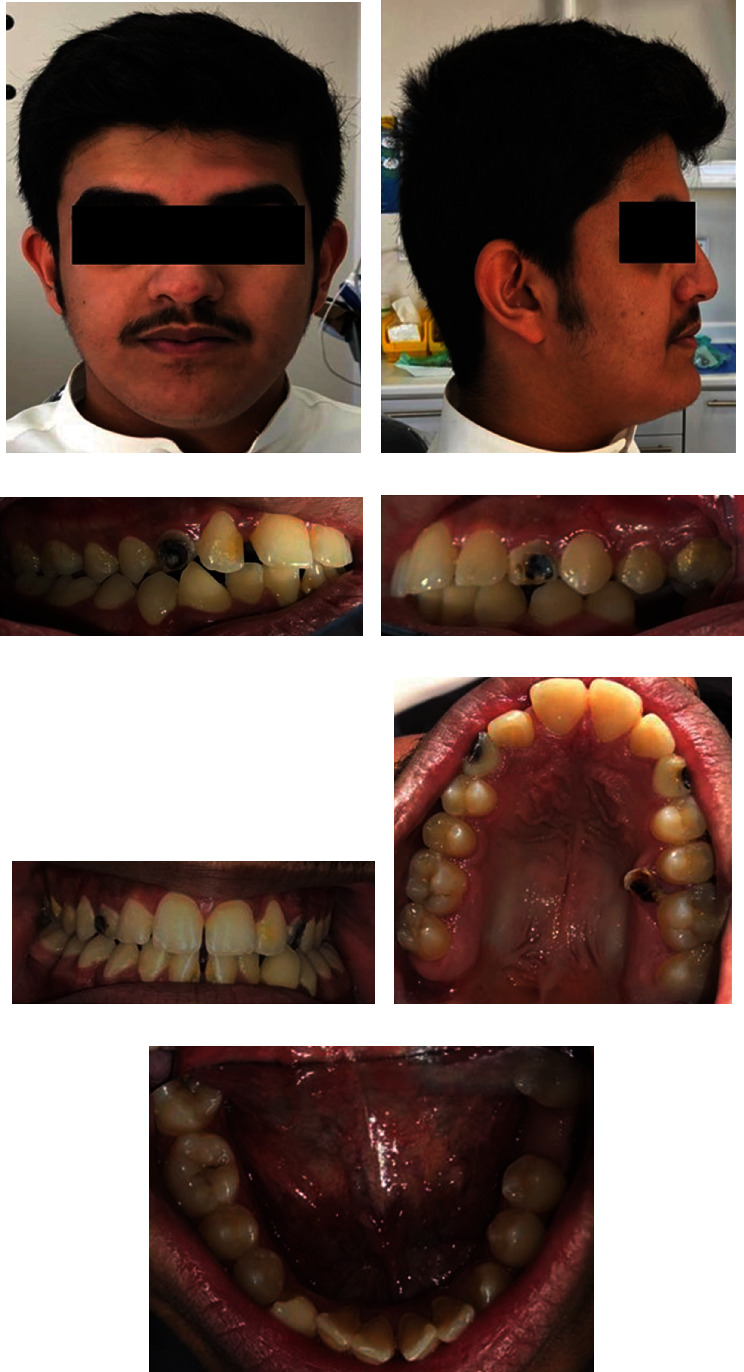
A clinical frontal view of the patient's face and neck (a), lateral profile view (b), intraoral lateral view of the patient's left side (c), IO right side (d), IO frontal view (e), IO upper occlusal view (f), and IO lower occlusal view (g).

The patient has a mesocephalic face with a concave profile, normal nasolabial angle, normal labiomental sulcus, symmetrical chin, and competent lips (Figures 1(a) and 1(b)).

### 2.3. Intraoral Examination

The examination showed poor oral hygiene with plaque and calculus accumulation on the lingual surface of the lower anterior teeth and multiple dental caries. The patient has a dental class III molar relationship, while canines' relationship was not applicable due to the missing permanent canines bilaterally, and the upper and lower midlines were centered. He has average vertical proportions, with 3 mm overjet and normal overbite. Moreover, the patient has retained maxillary primary canines, missing lower right first molar, upper mild spacing, and lower mild crowding (Figures 1(c)–1(g)).

### 2.4. Radiographic Assessment

**Figure 2:**
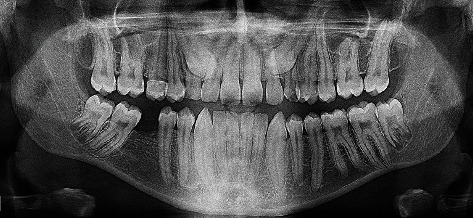
A panoramic radiograph showing the elongated roots of the teeth.

A panoramic radiograph was taken which incidentally revealed extremely long teeth with relatively wide roots in almost all dentition (Figure 2). The permanent maxillary canines were impacted with notably long roots.

The roots of the maxillary teeth appear superimposed on the maxillary sinus and extend to about the level of the middle meatus. Maxillary second molars showed very long root trunks.

**Figure 3:**
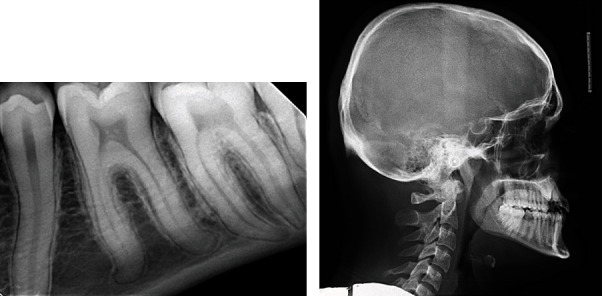
Periapical radiograph shows long roots of teeth #35, 36, 37 (a) and lateral cephalometric radiograph of the patient (b).

All mandibular teeth were long, especially the left second molar, which appears to be close to the inferior border of the mandible. The roots of mandibular third molars were developing in the inferior alveolar canals. The coronal portions of all teeth did not show any abnormalities in the aspect of shape or size. Further investigation to confirm the roots gigantism and to rule out any artifact was performed by taking periapical radiographs (Figure 3(a)). Due to the abnormal appearance of the roots, oculofaciocardiodental (OFCD) syndrome was included as a differential diagnosis. Thus, ophthalmology and cardiology teams were consulted for baseline assessment to rule out any association with OFCD syndrome. The ophthalmologic examination and investigation have included external eye examinations, visual acuity, extraocular muscles, visual fields, and intraocular pressure, while cardiology investigations have included CVS auscultation, ECG, and echocardiogram. Both investigations did not show any ophthalmological or cardiac abnormalities. Furthermore, there was no family history of ocular or cardiac abnormalities.

A lateral cephalometric radiograph was taken for orthodontic purposes to evaluate the skeletal relationship (Figure 3(b)). The lateral cephalometric analysis showed a skeletal class III jaw relationship with mild maxillary retrusion and normal mandibular position (ANB, -2.1°; SNA, 77.6°; SNB, 79.8°). The maxillary incisors were proclined while the mandibular incisors exhibited lingual inclination (U1-PP, 121.4°; L1-GoGn, 79.3°). All measurements were done compared to the normative values (Figure 3(b)).

**Figure 4:**
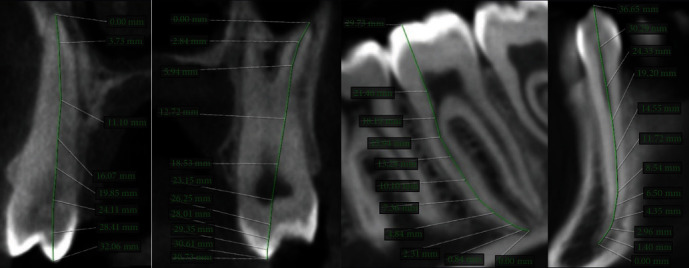
Sagittal section from cone beam computed tomography (CBCT) showing tooth sizes with radiculomegaly.

Cone beam computed tomography (CBCT) Planmeca Romexis® (Romexis 5.2.0., Helsinki, Finland) dental radiographic software was used to measure the length of the patient's entire set of teeth. Measurements were made from the apex to the tip of the cusps in a slice orientation manner (Figure 4). It has generally been reported that the vertical lengths of teeth share similar averages [19]. In this case, periapical radiographs (PAs) and CBCT revealed a significant variation in length. Permanent upper second premolar was measured to be 32.06 mm compared to the maximum reported length of 26.4 mm, the permanent upper second molar was 30.73 mm compared to the maximum reported length of 26.4 mm, permanent lower canine was 36.65 mm compared to the maximum reported length of 27.4 mm, and permanent lower second molar was 29.73 mm compared to the maximum reported length of 25.8 mm (Figure 4) [19].

## 3. Discussion

Radiculomegaly, or the marked elongation of the dental roots, is a rare congenital anomaly that has seldom been reported in the literature. The canine is the most affected tooth. The mandibular canine's length in a typical adult ranges from 24.6 to 27.4 mm with an average of 26 mm; therefore, a tooth is considered elongated when it is more than that ^19^. Moreover, the apices of the canines do not close until adulthood, and mandibular roots with radiculomegaly often closely approach the lower border of the mandible [13]. Hayward was the first to describe radiculomegaly in 1980 [5]. Then, two more cases of radiculomegaly were described in the literature after Hayward's report [6, 7]. Only a few cases of nonsyndromic/nonfamilial radiculomegaly have been reported in the literature since the description of OFCD in 1996, and all of them are not related to OFCD syndrome [8, 12, 18]. Similar to our case, the patient reported by Kemoli and Junior presented with a dental class III molar relationship [12]. Likewise, Al-Obaida noted that the patient in his case has a protruded mandible [18]. However, both cases reported a V-shaped maxilla in their patients. Unlike our patient, who presented with a U-shaped maxillary arch [12, 18]. It is worth mentioning that crowding in the upper anterior segment was noted in the comparable cases. While our patient presented with an upper mild spacing [12, 18]. Interestingly, radiculomegaly was found rarely in males prior to our report [6, 11, 16–18]. OFCD syndrome is an inherited disorder with a dominant X-linked pattern with potential lethality of male hemizygotes that may happen in the early stages of embryonic development even before pregnancy is observed [11, 20]. The cause of the disorder is believed to be a truncated mutation in the BCOR (BCL-6 interacting corepressor) gene [21–24]. All published reports about OFCD syndrome patients have been females and rarely found in males, and none of them were having OFCD according to the literature [6, 11, 16–18]. Among the male cases, one case was an OFCD syndrome patient's brother [11], one was born with Cockayne syndrome [17], the third case was a report showing root elongation of an extracted tooth without related primary clinical data [6], the fourth case was diagnosed with Klinefelter syndrome [16], and the last case was a nonsyndromic generalized radiculomegaly in a Saudi individual [18]. Wilkie and Chambers, in a case of a mother and her daughter, reported that before the syndromic daughter was born, 2 miscarriages happened to the mother [7]. The authors suggested that the dominance of the mutated X chromosome may have caused the abortion, which is considered fatal in males [4, 8, 9]. Hedera and Gorski reported that having a skewed pattern of X chromosome methylation and a nonrandom inactivation pattern are found in patients with OFCD syndrome after they were confirmed by a thorough genetic investigation [25]. The authors indicated that in other X-linked conditions such as incontinentia pigmenti and focal dermal hypoplasia, a similar incident of male lethality was reported [25]. Although, in the investigated family and in other reported cases, they noted no indication of spontaneous male abortion [25]. Radiculomegaly of a tooth is mostly observed with other dental, ocular, facial, and cardiac anomalies, and the condition is called oculofaciocardiodental syndrome (OFCD) [11]. The phenotypic spectrum of OFCD includes mostly microphthalmia, microcornea, secondary glaucoma, and congenital cataract of the eyes [26–28]. The facial dysmorphic features are often elongation of a narrow face with a high nasal bridge and broadening of the nasal tip with separated cartilages, long philtrum, and cleft palate [26–28]. The cardiac symptoms are mainly atrial septal defect, ventricular septal defect, and mitral valve prolapse [26–28]. Syndactyly of toes 2-3, septate vagina, and sensorineural hearing loss are less frequently related manifestations [26–28]. In the present case, although the patient reported a dental history of overretained primary maxillary canines and impaction of the permanent ones in addition to the extraction of the permanent mandibular right first molar, he did not report any other medical problems. No personal or family history of ocular or cardiac abnormalities was observed after examining the patient. The absence of typical characteristics found in OFCD syndrome patients makes the diagnosis unlikely for the present patient. So far, root gigantism of all teeth from incisors to molars was documented in a few reports [11, 18, 27, 29]. Here, we report a new instance of generalized radiculomegaly of permanent teeth and the association of OFCD syndrome with dental challenges [19]. In endodontic treatment, poor prognosis is usually in teeth with extraordinary long roots and open apices [14, 30]. Besides, accessing the root canal system surgically should be avoided in treating lower teeth due to the flap size required to reach the apex that can damage critical anatomic structures such as the inferior alveolar nerve [30]. Although root canal therapy is difficult in teeth with unusual lengths, special endodontic methods can be used in treating such cases [14, 30]. In cases with very deep caries, the immediate treatment of them and the use of vital pulp therapy make it possible to preserve the function of the tooth and to avoid complicated endodontic procedures [30]. Maden et al., 2010, and Barthelemy et al., 2001, suggested that devitalization and early endodontic intervention to induce apical closure can be effective as preventive procedures for gigantic roots [14, 31]. Careful periodical dental examination and oral hygiene instructions in patients with radiculomegaly are essential to prevent dental caries and subsequent root canal treatment [30]. Moreover, root gigantism may complicate orthodontic treatment and would extend the treatment time. However, alignment of the dental arches can be achieved using light forces to minimize the risk of ankylosis or shortening of the roots [32]. Uribe et al., 2011, showed that teeth with radiculomegaly can be repositioned with traction on skeletal anchorage without causing ankylosis [33]. Sakaguchi et al., 2012, proved that teeth with long roots in patients with skeletal problems can be treated effectively with surgical orthodontic treatment with a light force so as not to cause ankylosis [34]. Likewise, the large size of the roots entails difficulties that make the extraction challenging because of the great amount of resistance during the removal [5]. Finally, this report demonstrates the important role of dental professionals in screening clinics to recognize unusual incidental findings which can be essential in providing information for the diagnosis of OFCD syndrome.

## 4. Conclusion

Radiculomegaly is rarely found as a nonsyndromic/nonfamilial generalized abnormality in male gender that is not related to OFCD syndrome, as reported in this patient. Dental professionals need to be informed of the clinical and radiographic features of different dental anomalies, especially radiculomegaly, as it is considered one of the significant characteristics of OFCD syndrome.
